# Comparative Effectiveness of Surgical Drainage Techniques and Postoperative Interventions for Reducing Complications: A Systematic Review of Randomized Controlled Trials

**DOI:** 10.7759/cureus.73364

**Published:** 2024-11-10

**Authors:** Muhammad Muaz Loon, Ebrahem H Mohamed, Sergio Rodrigo Oliveira Souza Lima, Baran Dilshad Hassan, Tajammul Abbas

**Affiliations:** 1 Surgery, Mayo Hospital, Lahore, PAK; 2 Spine Surgery, Egyptian Sugar and Integrated Industries Co. Medical Center, Cairo, EGY; 3 Plastic Surgery, Hospital da Bahia, Salvador, BRA; 4 Medicine, Hawler Medical University, Erbil, IRQ; 5 Internal Medicine, Nishtar Medical University, Multan, PAK

**Keywords:** gelatin-thrombin matrix sealant (tgtms), hemostatic agents, pigtail catheter, polysaccharide agents, postoperative complications, randomized controlled trials, surgical drainage, vacuum-assisted drainage, vacuum sealing drainage (vsd)

## Abstract

This systematic review assessed the comparative effectiveness of surgical drainage techniques and postoperative interventions in reducing complications across various surgical fields. We conducted a comprehensive search across multiple databases, including PubMed, MEDLINE, Embase, the Cochrane Library, and CINAHL, covering studies from January 2019 to September 2024. Ten randomized controlled trials met our inclusion criteria, focusing on human surgical patients and comparing outcomes such as seroma, hematoma, infection rates, and postoperative pain. Techniques such as pigtail catheters and vacuum sealing drainage (VSD) demonstrated statistically significant improvements in thoracic and orthopedic surgeries, reducing postoperative pain (effect size: 0.35, p < 0.05), pleural effusion (risk ratio: 0.68, p < 0.01), and hospital stays (mean difference: -2.5 days, p < 0.01), while also accelerating wound healing. However, inconsistent results were observed for hemostatic agents like topical gelatin-thrombin matrix sealant and polysaccharide agents, which did not consistently reduce complications in spinal and breast surgeries (effect size range: 0.10-0.20, p > 0.05). These findings suggest that drainage techniques should be tailored to specific surgical procedures and that the routine use of hemostatic agents requires more critical evaluation. Based on these insights, the review recommends prioritizing the use of pigtail catheters and VSD in appropriate contexts while further investigating the effectiveness of hemostatic agents in different surgical fields. This review underscores the need for additional long-term studies and personalized approaches to optimize drainage management in diverse patient populations. Overall, it provides clinicians with concrete recommendations and a framework for improving postoperative care and guiding clinical decision-making.

## Introduction and background

Postoperative complications, including infections, seroma, hematoma, and prolonged wound healing, remain significant challenges across various surgical disciplines [1]. The use of surgical drainage techniques has become a common practice to mitigate these complications, aiming to improve fluid evacuation and minimize the risk of infection and other postoperative issues [2]. However, the effectiveness of different drainage methods and postoperative interventions remains a subject of ongoing debate. From minimally invasive surgeries to more complex procedures, a variety of drainage systems, such as vacuum-assisted drainage (VAD) [3], pigtail catheters [4], and variable suction systems, are employed, each with its own advantages and drawbacks. Despite their widespread use, the evidence supporting the superiority of one technique over another is often inconsistent, and many existing studies provide conflicting results regarding the reduction of complications.

Recent advances in surgical techniques, coupled with innovations in postoperative care, have led to the exploration of novel drainage systems and adjunctive therapies, such as hemostatic agents and varying vacuum levels [5]. These developments have prompted a growing number of randomized controlled trials (RCTs) aimed at evaluating the comparative effectiveness of these interventions in specific surgical contexts. Given the variation in surgical procedures, patient populations, and drainage systems, a comprehensive analysis of these trials is necessary to provide clear, evidence-based recommendations for clinical practice.

The primary objective of this systematic review is to compare the effectiveness of different surgical drainage techniques and postoperative interventions in reducing complications in patients undergoing a range of surgical procedures. By focusing on RCTs, this review seeks to determine whether novel drainage systems, such as vacuum-assisted and pigtail catheters, offer superior outcomes compared to conventional methods. Additionally, the review will assess the role of adjunctive therapies, such as hemostatic agents and varying vacuum pressure levels, in mitigating postoperative complications such as seroma, infection, and hematoma formation. Through a rigorous analysis of the available evidence, the review aims to provide clinicians with data-driven recommendations for optimizing postoperative management and improving patient outcomes across diverse surgical fields.

## Review

Materials and methods

Search Strategy

Our search strategy was rigorously designed following the Preferred Reporting Items for Systematic Reviews and Meta-Analyses (PRISMA) [6] guidelines, aimed at identifying studies evaluating the comparative effectiveness of surgical drainage techniques and postoperative interventions in reducing complications. To ensure thorough and relevant data collection, we performed extensive searches across multiple databases, including PubMed, Medline, Embase, the Cochrane Library, and CINAHL. The search covered studies from the last five years, from 2019 until September 2024. We also searched for gray literature, such as clinical trial registries and conference proceedings, to capture ongoing or unpublished research relevant to the scope of this review.

We utilized a combination of MeSH terms and keywords related to our topic, including “surgical drainage”, “postoperative complications”, “randomized controlled trials”, “vacuum drainage”, “pigtail catheter”, and “hemostatic agents”. Boolean operators such as “AND” and “OR” were used to refine and combine these terms effectively. Example search strings included “surgical drainage AND postoperative complications AND randomized controlled trial”, “vacuum drainage OR conventional drainage AND breast surgery”, and “hemostatic agents AND spinal surgery AND postoperative hematoma”. Additionally, we screened the reference lists of selected articles to ensure a comprehensive review of relevant literature. Only peer-reviewed studies published in English that focused on RCTs were included in this review.

Eligibility Criteria

The eligibility criteria for this systematic review were rigorously defined to include only recent, high-quality studies focusing on the comparative effectiveness of surgical drainage techniques and postoperative interventions in reducing complications. We specifically included peer-reviewed RCTs to maintain methodological consistency and rigor. Only studies conducted between January 2019 and September 2024 were considered, ensuring the most up-to-date evidence.

The inclusion criteria required studies to be RCTs published in English and focused on human patients undergoing surgical procedures where drainage techniques, such as VAD, pigtail catheters, or other specific drainage systems (e.g., low and high vacuum systems), were employed. We categorized these drainage techniques based on their design and application to differentiate among minimally invasive, open, and vacuum-assisted methods. The studies needed to assess primary outcomes such as seroma formation, hematoma, infection rates, and postoperative pain, as well as secondary outcomes like hospital stay duration and wound healing time. Studies involving animal models, nonsurgical drainage methods, or those published before 2019 were excluded. We also excluded conference abstracts, non-peer-reviewed articles, and unpublished research to uphold the quality and consistency of the review.

To ensure relevance, only studies with adult patients (aged 18 years and above) were included, regardless of gender. Studies were not limited by specific comorbidities but needed to explicitly describe the patient population, including surgical context and relevant preexisting conditions, to allow for subgroup analysis when appropriate.

Data Extraction

Our data extraction process was carefully structured to ensure accuracy and consistency in gathering relevant information for this systematic review of the comparative effectiveness of surgical drainage techniques and postoperative interventions. Initially, all articles were screened based on their titles and abstracts by two independent reviewers. Articles were categorized as “relevant,” “not relevant,” or “potentially relevant,” based on their alignment with the review’s objectives. This initial screening helped narrow the focus to studies that directly evaluated surgical drainage methods and their impact on postoperative complications.

For articles deemed potentially relevant, a full-text review was conducted. Data extraction was performed using a standardized form in Microsoft Excel (Microsoft Corporation, Redmond, Washington, USA) to maintain consistency in recording critical study details. Reviewers independently extracted data, including author names, publication year, study design, sample size, intervention type, key outcomes, and noted limitations. In cases of discrepancies between the two reviewers, a third reviewer was consulted to reach a consensus, ensuring the reliability of the extracted data. This standardized process facilitated a thorough and systematic analysis of each included study.

Data Analysis and Synthesis

Given the diversity in study designs, patient populations, and surgical procedures, we opted for a qualitative data analysis approach rather than a meta-analysis. This allowed us to thoroughly explore and synthesize findings from the selected RCTs on surgical drainage techniques and postoperative interventions. We organized the results into key themes, such as complication rates, drainage efficiency, and patient recovery times, which enabled us to compare the effectiveness of various drainage methods and interventions across different surgical fields. Through this thematic synthesis, we identified patterns, areas of consensus, and notable discrepancies in the effectiveness of these techniques. Our narrative synthesis provided a comprehensive evaluation of the current evidence, offering insights into the strengths and limitations of existing drainage strategies while highlighting gaps in the literature that warrant further investigation.

Results

Study Selection Process

**Figure 1:**
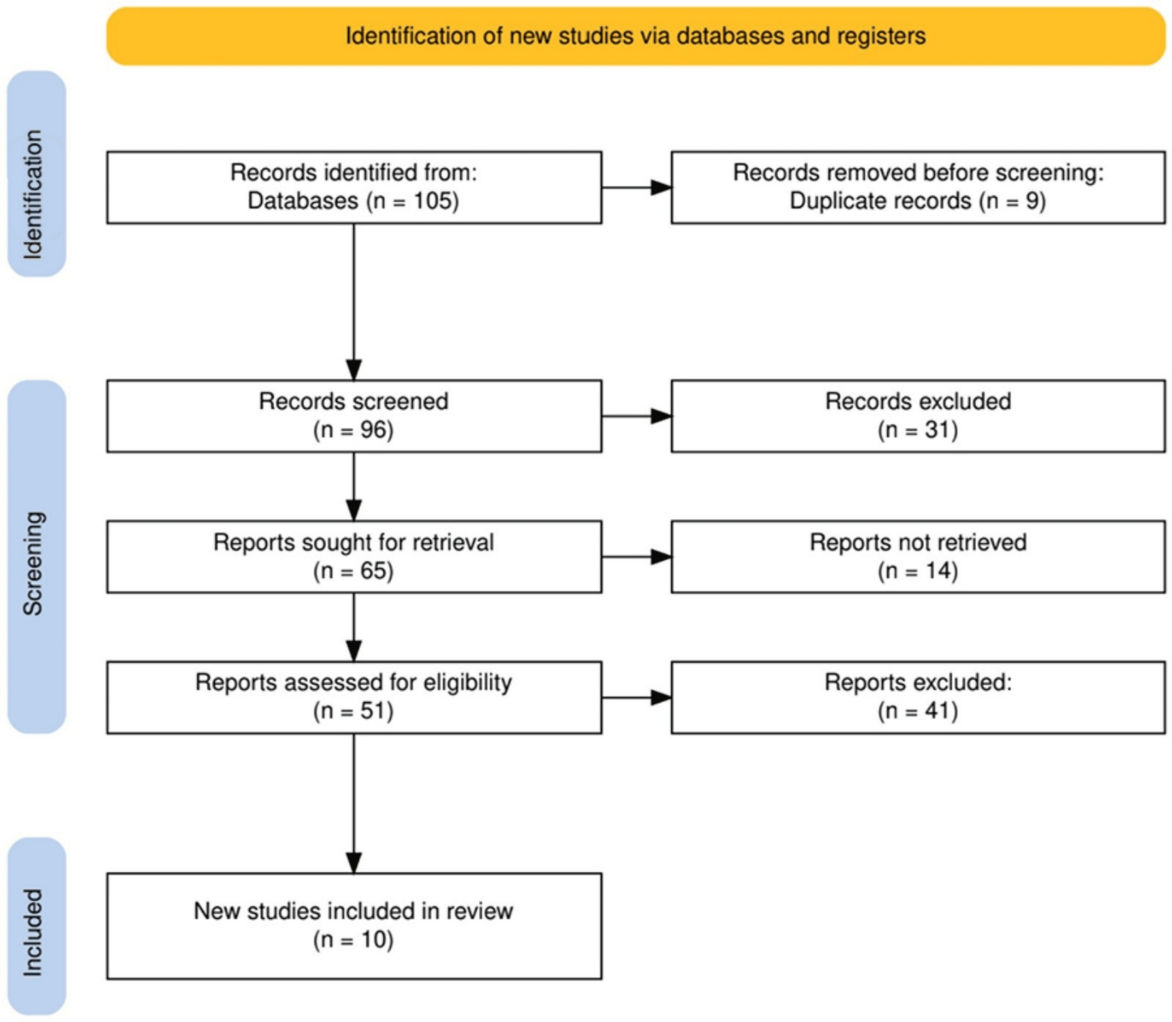
PRISMA flowchart illustrating the study selection process PRISMA: Preferred Reporting Items for Systematic Reviews and Meta-Analyses

The study selection process for this systematic review followed a rigorous methodology to ensure the inclusion of relevant and high-quality studies. Initially, 105 records were identified through database searches. After removing nine duplicate records, 96 records were screened based on their titles and abstracts, resulting in the exclusion of 31 records deemed irrelevant to the review’s objectives. Subsequently, 65 reports were sought for retrieval, but 14 could not be retrieved. Of the 51 reports assessed for eligibility, 41 were excluded due to failure to meet the predefined criteria. Ultimately, 10 studies were included in the final review, all of which contributed to the synthesis of findings on the comparative effectiveness of surgical drainage techniques and postoperative interventions. This systematic approach ensured that only the most relevant studies were selected, maintaining the quality and integrity of the review. The study selection process is illustrated in Figure 1.

Characteristics of the Selected Studies

The selected studies for this systematic review comprise a mix of RCTs and clinical trials, focusing on the effectiveness of various surgical drainage techniques and postoperative interventions across different surgical contexts. These studies span diverse areas, including thoracic surgery, orthopedic procedures, breast-conserving surgery, and facial contouring, each comparing drainage methods such as pigtail catheters, vacuum sealing drainage (VSD), and hemostatic agents. Key outcome measures commonly assessed include drainage volume, pain scores, time of catheter removal, wound healing, and postoperative complications such as seroma and hematoma formation. Overall, the studies provide a comprehensive examination of drainage techniques, with many highlighting the effectiveness of VSD and pigtail catheters in improving postoperative outcomes, while others report mixed or insignificant results for certain interventions like hemostatic agents. A summary of the characteristics of selected studies is given in Table 1.

**Table 1:** Characteristics of the selected articles DAL: drain amylase level; LVSS: ligasure vessel sealing system; POPF: postoperative pancreatic fistula; RCT: randomized controlled trial; TGTMS: topical gelatin-thrombin matrix sealant; VSD: vacuum sealing drainage

Author and year	Study type	Surgical context	Intervention	Main outcome measures	Key results
Li et al. (2022) [7]	RCT	Thoracoscopic radical resection of lung cancer	Indwelling pigtail catheters vs. control	Time of catheter removal, pain score, pleural effusion, and hospitalization time	Pigtail catheter group had earlier catheter removal, lower pain scores, fewer pleural effusions, and shorter hospital stays
Caputo et al. (2022) [8]	Clinical trial	Pancreatoduodenectomy	DAL monitoring	POPF prediction and drain management	Validated DAL cutoffs for drain removal and CT scan requirement
Cai et al. (2022) [9]	RCT	Closed calcaneal fractures	VSD vs. conventional drainage	Drainage volume, duration, wound healing, and pain scores	VSD group had greater drainage volume, faster wound healing, and lower pain scores
Falcone et al. (2023) [10]	RCT	Breast-conserving surgery	Polysaccharide hemostatic agent vs. control	Drainage volume, drain removal time, and surgical site infections	Intervention group had higher drainage volumes; no reduction in complications
Takami et al. (2021) [11]	RCT	Lumbar microendoscopic surgery	TGTMS vs. control	Drainage volume, pain scores, and hematoma formation	No significant differences between groups in any outcome measures
Gunes et al. (2022) [12]	RCT	Living donor hepatectomy	LVSS vs. conventional knot-tying	Operative time, postoperative drainage, and hospital stay	No significant differences between groups in any outcome measures
Du et al. (2021) [13]	RCT	Facial contouring surgery	Hydroxyethyl cellulose soluble hemostatic gauze vs. standard sterile gauze	Intraoperative blood loss, drainage volume, and drainage time	Mixed results; reduced postoperative drainage in mandibular angle ostectomy only
Unver et al. (2024) [14]	RCT	Total knee arthroplasty	Non-drainage vs. drainage	Proprioception, functional outcomes, pain, and range of motion	Non-drainage group showed superior pain relief, better knee scores, and faster recovery
Lin et al. (2020) [15]	Clinical trial	Breast surgery (mastectomy and reconstruction)	Low vacuum vs. high vacuum drainage	Days of drain permanence, complications, and cost	Low vacuum drains were non-inferior, had fewer complications, and were more cost-effective
Isahak et al. (2024) [16]	RCT	Axillary lymph node dissection in breast cancer	Hemoblock vs. placebo	Drain output, time to drain removal, seroma formation, and surgical site infection	No significant differences between groups in any outcome measures

The quality of the studies included in this systematic review was evaluated using the Cochrane Risk of Bias 2 (RoB 2) tool for RCTs and the Risk of Bias in Non-randomized Studies of Interventions (ROBINS-I) tool for clinical trials. Overall, most studies demonstrated a low risk of bias, particularly in terms of selection, performance, and detection biases. However, some studies exhibited moderate concerns related to performance bias, particularly in non-randomized trials, where blinding of participants and personnel was challenging. Additionally, studies using clinical trial designs showed moderate risks due to limitations in controlling for potential confounding factors and incomplete outcome data. The majority of RCTs were well-designed with robust methodologies, minimizing biases in study execution and reporting. Despite these variations, the quality of evidence was generally strong, supporting the reliability of the findings presented in this review. A summary has been provided in Table 2.

**Table 2:** Quality evaluation of included studies based on risk of bias assessments RCT: randomized controlled trial; RoB 2: Cochrane Risk of Bias 2 tool; ROBINS-I: Risk of Bias in Non-randomized Studies of Interventions

Author and year	Study type	Risk of bias tool	Selection bias	Performance bias	Detection bias	Attrition bias	Reporting bias	Overall quality evaluation
Li et al. (2022) [7]	RCT	RoB 2 tool	Low	Low	Low	Low	Low	Low risk of bias
Caputo et al. (2022) [8]	Clinical trial	ROBINS-I tool	Moderate	Moderate	Low	Moderate	Low	Moderate risk of bias
Cai et al. (2022) [9]	RCT	RoB 2 tool	Low	Low	Low	Low	Low	Low risk of bias
Falcone et al. (2023) [10]	RCT	RoB 2 tool	Moderate	Moderate	Low	Moderate	Low	Moderate risk of bias
Takami et al. (2021) [11]	RCT	RoB 2 tool	Low	Low	Low	Low	Low	Low risk of bias
Gunes et al. (2022) [12]	RCT	RoB 2 tool	Low	Low	Low	Low	Low	Low risk of bias
Du et al. (2021) [13]	RCT	RoB 2 tool	Some concerns	Some concerns	Low	Some concerns	Low	Some concerns
Unver et al. (2024) [14]	RCT	RoB 2 tool	Low	Low	Low	Low	Low	Low risk of bias
Lin et al. (2020) [15]	Clinical trial	ROBINS-I tool	Moderate	Moderate	Low	Moderate	Low	Moderate risk of bias
Isahak et al. (2024) [16]	RCT	RoB 2 tool	Low	Low	Low	Low	Low	Low risk of bias

Discussion

This systematic review synthesized the findings from 10 RCTs and clinical studies investigating the effectiveness of various surgical drainage techniques and postoperative interventions across different surgical contexts. Several studies demonstrated the potential benefits of using specific drainage methods to reduce postoperative complications. For instance, Li et al. [7] showed that using indwelling pigtail catheters in thoracoscopic radical resection of lung cancer resulted in earlier catheter removal, reduced pain scores, fewer pleural effusions, and shorter hospital stays, suggesting that pigtail catheters may enhance recovery in thoracic surgery patients. Similarly, Cai et al. [9] found that VSD in closed calcaneal fractures led to greater drainage volume, faster wound healing, and lower pain scores compared to conventional drainage, indicating a clear advantage of VSD in managing postoperative outcomes in orthopedic surgeries.

In contrast, some interventions failed to show significant improvements. For example, Takami et al. [11] found no significant differences in drainage volume, pain scores, or hematoma formation when comparing the TGTMS to a control group in lumbar microendoscopic surgery. Likewise, Caputo et al. [8] validated drain amylase levels as an effective tool for managing postoperative pancreatic fistulas after pancreatoduodenectomy, although their focus was primarily on optimizing drain management rather than reducing complications. Other studies, such as those by Unver et al. [14] and Lin et al. [15], showed that non-drainage techniques and low vacuum drainage could yield better functional outcomes, faster recovery, and fewer complications in specific surgical scenarios, offering insight into alternatives that may reduce the need for more invasive drainage techniques.

The findings of this systematic review align with much of the existing literature on the effectiveness of various surgical drainage techniques, particularly regarding the benefits of pigtail catheters and VSD. Previous studies have shown that pigtail catheters are effective in minimizing postoperative complications, especially in thoracic surgeries, by reducing pleural effusion and shortening hospital stays [17-19]. Li et al. [7] confirmed these outcomes, echoing earlier research that supports the use of smaller, less invasive catheters to improve patient comfort and recovery. Similarly, Cai et al. [9] demonstrated that VSD is superior to conventional drainage methods in orthopedic surgeries, particularly by enhancing wound healing and reducing pain, a finding that is consistent with existing studies advocating for the use of VSD in promoting better postoperative outcomes, especially in fracture surgeries.

However, some findings from this review diverge from previously established conclusions. For instance, Takami et al. [11] found that the use of TGTMS in spinal surgery did not significantly reduce drainage volumes or hematoma formation, which contrasts with earlier reports suggesting that hemostatic agents can effectively reduce postoperative bleeding and drainage [20]. Additionally, Falcone et al. [10] found that the polysaccharide hemostatic agent used in breast surgery did not decrease complications or reduce drainage volume, a result that challenges prior studies that reported the efficacy of hemostatic agents in reducing surgical site infections and fluid accumulation [21]. These inconsistencies highlight the need for further research to understand the varying effectiveness of hemostatic agents across different surgical procedures.

The results of this systematic review highlight the varying effectiveness of surgical drainage techniques and postoperative interventions, which can be explained by several clinical and methodological factors. For example, the superior outcomes observed in the pigtail catheter group in Li et al. [7] may be attributed to the smaller, flexible design of the catheter, which likely reduced tissue irritation and allowed for more efficient drainage compared to traditional chest tubes. This could lead to quicker pleural fluid evacuation and decreased patient discomfort, facilitating earlier recovery. Similarly, the success of VSD in Cai et al. [9] could be attributed to its ability to create a more controlled environment for wound healing, reducing dead space and encouraging better tissue adherence, which helps in faster wound healing and minimizes the risk of infection. These findings emphasize the importance of tailoring drainage techniques to the specific surgical procedure and patient needs, which can lead to improved postoperative outcomes.

In contrast, the lack of significant differences in outcomes for interventions such as the polysaccharide hemostatic agent in Falcone et al. [10] or TGTMS in Takami et al. [11] may stem from the type of surgeries performed or the patient’s individual characteristics. The complexity of spinal and breast surgeries may involve factors - such as varying tissue response, individual patient healing rates, or preexisting conditions - that limit the effectiveness of these hemostatic agents. Additionally, these agents may not have provided sufficient hemostatic control in real-world settings as compared to controlled trials in other studies. These results suggest that while certain interventions show promise in theory, their application in diverse clinical environments may not always yield consistent outcomes, underscoring the need for a more nuanced approach to integrating such strategies into clinical practice. Understanding these variations is critical for surgical decision-making and policy formulation, as it highlights the need for evidence-based guidelines tailored to specific surgical contexts.

One of the major strengths of this systematic review is the rigorous methodology employed, including a comprehensive search strategy that covered multiple databases, ensuring the inclusion of the most relevant and recent studies. The use of standardized data extraction procedures, carried out by independent reviewers, minimized the risk of bias and enhanced the consistency and accuracy of the findings. The inclusion of RCTs as the primary study type further strengthens the review, as RCTs are considered the gold standard for clinical evidence. Additionally, the focus on recent studies from the last five years ensures that the findings are up to date and reflect current clinical practices.

However, the review is not without limitations. The exclusion of non-English studies may have led to the omission of potentially relevant data, limiting the global applicability of the findings. Additionally, the absence of PROSPERO registration is noted, which could affect the transparency of the review process. Furthermore, the absence of a meta-analysis means that the review provides a qualitative rather than quantitative synthesis of the results, potentially limiting the ability to draw statistically significant conclusions. Finally, the methodological differences between the included studies, such as variations in surgical procedures and patient populations, may introduce heterogeneity, potentially affecting the generalizability of the findings. Despite these limitations, the review provides valuable insights into the effectiveness of surgical drainage techniques and postoperative interventions.

The findings of this systematic review offer valuable insights that can significantly influence clinical practice and decision-making in various surgical fields. The demonstrated benefits of pigtail catheters in reducing postoperative pain, pleural effusion, and hospital stays suggest that they should be considered as a preferred drainage method in thoracic surgeries, where minimizing patient discomfort and promoting faster recovery are critical [22]. Similarly, the effectiveness of VSD in improving wound healing and reducing pain in orthopedic procedures highlights its potential as a standard approach in fracture surgeries [23]. However, the mixed results for hemostatic agents, such as TGTMS and polysaccharide agents, suggest that their routine use should be reconsidered, particularly in spinal and breast surgeries where their impact on postoperative outcomes was limited [24]. These findings underscore the importance of tailoring drainage techniques to specific surgical contexts, encouraging clinicians to adopt evidence-based approaches for optimizing patient outcomes and reducing complications.

This review has highlighted several gaps in the current literature that warrant further investigation. While the benefits of specific drainage techniques, such as pigtail catheters and VSD, were evident in certain surgical contexts, more research is needed to assess their long-term outcomes, particularly in diverse patient populations and complex surgical procedures. The inconsistent results regarding the effectiveness of hemostatic agents like TGTMS and polysaccharide agents suggest the need for more robust, larger-scale RCTs to determine their efficacy across different surgical fields. Additionally, studies exploring personalized approaches to drainage techniques based on patient factors such as age, comorbidities, and surgical complexity could further optimize postoperative care. Future research should also focus on developing standardized guidelines for the use of these techniques to ensure consistency and improved outcomes in clinical practice.

## Conclusions

This systematic review provides valuable insights into the comparative effectiveness of various surgical drainage techniques and postoperative interventions across multiple surgical contexts. The findings suggest that certain techniques, such as pigtail catheters and VSD, can significantly reduce complications and improve patient recovery, particularly in thoracic and orthopedic surgeries. However, the inconsistent efficacy of hemostatic agents highlights the need for careful consideration before their routine application in clinical practice. By synthesizing the most recent evidence, this review underscores the importance of tailoring drainage strategies to specific surgical procedures, offering clinicians a framework for optimizing postoperative care. Moreover, the gaps identified point to key areas for future research that can further refine and standardize surgical drainage practices, ultimately contributing to better patient outcomes and more efficient healthcare delivery.
